# Appendicite aigue non compliquée: y a-t- il une place pour le traitement conservateur

**DOI:** 10.11604/pamj.2015.21.144.6045

**Published:** 2015-06-23

**Authors:** Ahmed El Khader, Mohamed Lahkim, Rachid El Barni, Abdessamad Achour

**Affiliations:** 1 Service de Chirurgie Générale, Hôpital Militaire Avicenne, Marrakech, Maroc

**Keywords:** Appendicite aigue, traitement conservateur, antibiotique, acute appendicitis, Conservative treatment, antibiotic

## Abstract

Le but de cette étude a été d’évaluer l'efficacité de l'antibiothérapie seule dans le traitement des appendicites aigues non compliquées. C'est une étude prospective, intéressant 68 patients ayant eu une appendicite aigue simple, de confirmation radiologique, traités par l'amoxicilline associée à l'acide clavulanique pendant 10 jours. L'appendicectomie a été réalisée en cas d'aggravation ou en cas de non amélioration au bout de 48heures de traitement. Le traitement conservateur a été efficace dans 82,35% avec une résolution complète des symptômes chez 56 patients. Les 12 cas restants (17,65%) ont subit une appendicectomie. l'appendicite a été gangréneuse dans 8 cas et phlegmoneuse dans 4 cas. Cinq des 56 patients, qui ont bien évolué sous traitement conservateur, ont été réadmis et opérés pour récidive, soit 8,9%. Deux cas ont eu une appendicite compliquée. L'appendicectomie reste le traitement de référence pour l'appendicite aigue, mais le traitement antibiotique peut être proposé en première intension à des patients présentant une appendicite aigue non compliquée.

## Introduction

Les appendicites aigues constituent l'indication chirurgicale d'urgence la plus fréquente dans le monde pour les douleurs abdominales aigues. L'appendicectomie, à ciel ouvert ou par voie laparoscopique, est le traitement de référence, mais elle reste non dénuée de complications, notamment les occlusions sur bride. Des données ont poussé, ces dernières années, plusieurs équipes à tenter un traitement conservateur de l'appendicite aigue non compliquée (AANC) en se basant sur l'antibiothérapie seule [1–4]. L’étiologie de l'appendicite: Les appendicites par obstruction de l'appendice, conduisant à la gangrène et à la perforation, sont en réalité les plus rares (10%). Les appendicites phlegmoneuses non liées à l'obstruction sont les plus fréquentes; l’évolution favorable sous antibiothérapie seule des autres infections viscérales, notamment les salpingites et les diverticulites non compliquées; le taux non négligeable d'appendicectomie, réalisée pour un appendice normal; le nombre de réadmission après chirurgie. A dix ans, 21% des patients ayant subit une appendicectomie sont réhospitalisés pour une suspicion d'occlusion sur bride et 2,7% sont réopérés. Notre étude a pour but d’évaluer la sureté et l'efficacité de l'antibiothérapie seule dans le traitement des AANC et d'essayer de montrer que l'appendicectomie n'est pas leur seul traitement.

## Méthodes

C'est une étude prospective, réalisée dans le service de chirurgie générale de l'hôpital militaire Avicenne de Marrakech, pour une période de 28 mois, allant du septembre 2010 au décembre 2012, intéressant 68 patients ayant eu une appendicite aigue simple, qui ont fait l'objet d'un protocole thérapeutique consistant en une antibiothérapie par voie veineuse à base d'amoxicilline associée à l'acide clavulanique pendant 48 heures avec un relai par voie orale pendant 8 jours. L'appendicectomie a été réalisée en cas d'aggravation ou en cas de non amélioration au bout de 48 heures. Tous les patients, admis aux urgences pour suspicion d'appendicite, ont fait l'objet d'un bilan clinique, biologique et radiologique pour une possible inclusion dans l’étude. Le diagnostic de l'appendicite aigue non compliquée a été retenu en présence de l'ensemble des critères suivants: une douleur de la fosse iliaque droite; une fièvre ou une hyperleucocytose; un diamètre de l'appendice > 6mm à l’échographie ou au scanner, en absence de signes de complications (plastron, abcès, épanchement localisé ou diffus). Une fois le diagnostic d'appendicite aigue non compliquée a été retenu, le patient a été informé du protocole et invité à y participer. Tous les patients ont été suivis en consultation et vus 15 jours, un mois et un an après leur sortie de l'hôpital.

## Résultats

**Figure 1:**
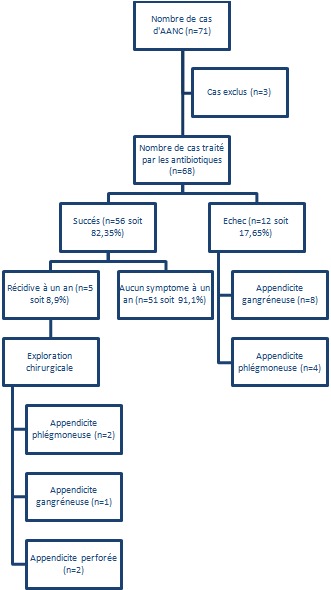
Schéma résumant les résultats, obtenus dans notre étude, du traitement antibiotique dans les appendicites aigues non compliquées

Entre septembre 2010 et décembre 2012, soixante et onze patients, ayant eu une appendicite aigue non compliquées, ont été pris en charge dans notre service. Après les avoir informé, soixante huit ont été inclus dans l’étude. Les 3 patients ayant refusé le traitement conservateur, ont été opérés. Quarante six patients (67,65%) ont été de sexe masculin et vingt deux (32,35%) de sexe féminin. L’âge de nos patients a été entre 16 et 59 ans avec une moyenne d’âge de 34, 36. La symptomatologie clinique a été faite essentiellement d'une douleur abdominale aigue (68 cas soit 100%), des nausées et/ou vomissements (58 cas soit 85,29%). La température à été comprise entre 37°C et 38°C dans 48 cas (70,5%) et supérieure à 38°C dans 20 cas (29,5%). L'examen clinique a objectivé une défense de la fosse iliaque droite dans 29 cas (42,6%) et une simple sensibilité abdominale dans 39 cas (57,4%). Le taux des leucocytes a été compris entre 3400 et 23700 avec une moyenne de 13800. L’échographie abdominale a été réalisée chez tous nos patients. Elle a confirmé le diagnostic dans 57 cas (83,8%). Le scanner a permis de faire le diagnostic dans les 11 cas restants (16,2%). Le diamètre de l'appendice à l'imagerie a été compris entre 7,6 mm et 13 mm avec une moyenne de 9,34 mm. L'imagerie a montré également une infiltration de la graisse périappendiculaire dans 22 cas (32,35%) et la présence d'un stercolithe dans 5 cas (7,35%). Tous les patients ont été mis sous amoxicilline associée à l'acide clavulanique par voie veineuse à une dose quotidienne de 3 g, répartie en 3 prises, pendant une période de 48 heures. L'aggravation ou la non amélioration au bout de 48heures a été considérée comme un échec du traitement médical, conduisant à une appendicectomie. Dans les cas contraires, l'antibiothérapie a été poursuivie par voie orale pendant 8 jours. Le traitement conservateur a été efficace dans 82,35% avec une résolution complète des symptômes chez 56 patients. Les 12 cas restants (17,65%) ont subit une appendicectomie. l'appendicite a été gangréneuse dans 8 cas et phlegmoneuse dans 4 cas. La durée d'hospitalisation est allée de 2 à 7 jours avec une moyenne de 3,7 jours. Tous les patients ont été suivis pendant une année. Les cas, qui n'ont pas été au rendez-vous, ont été contactés par téléphone. Ils ont tous déclaré qu'ils n'ont eu aucune symptomatologie. Cinq des 56 patients, qui ont bien évolué sous traitement conservateur, ont été réadmis et opérés pour récidive, soit 8,9%. Tous vus entre le 3éme et le 12^ème^mois de leur première poussée. Deux cas ont eu une appendicite compliquée (un abcès appendiculaire et une péritonite généralisée) (Figure 1).

## Discussion

L'appendicectomie est considérée depuis plus d'un siècle comme traitement standard de l'appendicite aigue, permettant d'obtenir la guérison et d’éviter l’évolution vers des complications plus graves à savoir la perforation et la diffusion péritonéale et générale de l'infection [5]. Si l'appendicectomie est considérée comme un traitement radical avec de bons résultats, elle n'est pas dénuée de complications. Ces complications qui sont représentées essentiellement par l'infection de la paroi, les suppurations profondes et l'occlusion sur bride, varient en fréquence entre l'appendicectomie conventionnelle et laparoscopique. Le taux global de ces complications est respectivement de 8,7% et 11,1% [6–8]. Durant ces dernières années, plusieurs études ont été publiées, dont l'objectif a été d’évaluer l'efficacité de l'antibiothérapie seule, comme traitement conservateur des appendicites aigues non compliquées, permettant ainsi d’éviter l'appendicectomie. La plupart des auteurs ont rapportés des études comparatives entre l'antibiothérapie et l'appendicectomie. Le taux de récidives a été comparé au taux de complications graves après l'appendicectomie. En 2006, Styrud a réalisé un essai randomisé et a traité des appendicites aigues non compliquées, soit par appendicectomie (n = 124), soit par antibiotiques (n = 128). Dans le groupe chirurgie, il y'a eu 14% des complications. Le traitement antibiotique a été efficace dans 86% des cas. Le taux de récidives à un an a été de 14% [9]. En 2009, Hansson a rapporté une étude randomisée et comparatives entre deux groupes de patients ayant une AANC diagnostiquée cliniquement et si nécessaire en s'aidant d'une échographie ou un scanner. L'antibiothérapie a été efficace dans 90,8% des cas. Le taux de récidives à un an a été de 13,9%. un tiers est survenu dans les 10 jours après la sortie de l'hôpital. Les deux tiers, 3 et 6 mois après. Il y'a eu trois fois plus de complications majeures dans le groupe chirurgie [10]. En 2011, Vons a rapporté une étude randomisée, multicentrique, comparant aussi l'appendicectomie à l'antibiothérapie. Le diagnostic d'AANC a été fait par le scanner. L'efficacité des antibiotiques à un an a été de 68% [11].

La quasitotalité de ces études a des limites avec présence de facteurs de confusion qui pourraient avoir une influence sur les résultats. L'inclusion a été souvent basée sur des données clinicobiologiques [9, 12, 13] et rarement sur la confirmation radiologique [11]. Le type et la durée du traitement antibiotique varient d'une étude à l'autre. La comparaison des complications entre les deux groupes a été un sujet de débat, ainsi par exemple la récidive ne peut se voir dans le groupe chirurgie, de même l'infection de la paroi pour le groupe antibiotique. La durée de suivi d'un an a été considérée comme insuffisante, ainsi les récidives peuvent survenir au-delà d'une année [5, 11, 14]. Notre travail a été réalisé dans le but d’évaluer la sureté et l'efficacité de l'antibiothérapie seule dans le traitement des AANC, tout en essayant d’éviter certaines limites reprochées aux autres études. Pour cette raison notre étude n'a pas été comparative et l'inclusion a été basée sur la confirmation radiologique et l'accord du patient. En comparant nos résultats avec les données de la littérature, on constate qu'on a eu un peu plus d’échec et moins de récidives à un an. Mais le nombre de patients à qui on a pu éviter l'intervention chirurgical est concordant avec les autres études, ainsi l'efficacité du traitement conservateur à un an, qui varie de 60% à 85% dans la littérature, a été de 75% dans notre étude (Tableau 1) [9–13]. Dans les deux tiers des cas qui ont subit l'appendicectomie après l’échec du traitement antibiotique, l'appendice a été gangrené. Le stercolithe a été présent dans un tiers des cas. Dans deux des cinq cas de récidive, l'appendice a été perforé avec présence de stercolithe. Nous pensons, comme certains auteurs, que la présence de stercolithe est un facteur de risque complications et de récidive [11, 15]. L'identification d'autres facteurs sera d'un grand intérêt pour le chirurgien afin de sélectionner des patients pour un traitement conservateur ou chirurgical.

**Tableau 1:** Résultats des principales études concernant l'efficacité du traitement antibiotique seuldans les appendicites aigues non compliquées

Auteurs (Année)	Nombre de patients	Mode de diagnostic	Taux d’échec	Taux de récidive à un an	L'efficacité à un an
Eriksson(1995)	20	Clinique	5%	35%	60%
Styrud(2006)	128	Clinique	14%	14%	74%
Hansson(2009)	106	Clinique±Echo ou TDM	8,2%	13,9%	78%
Malik(2009)	40	Score d'Alvaro	5%	10,5%	85%
Vons(2011)	120	TDM	12%	26%	68%
Notre étude	68	Echographie±TDM	17,65%	8,9%	75%

## Conclusion

L'appendicectomie reste le traitement de référence pour l'appendicite aigue, mais le traitement antibiotique peut être proposé en première intension à des patients présentant une AANC. Ce traitement conservateur permet d’éviter la chirurgie et ses complications à plus des deux tiers des patients, tout en sachant que son échec n'augmente pas la morbidité. L'appendicectomie reste le traitement de référence pour l'appendicite aigue, mais le traitement antibiotique peut être proposé en première intension à des patients présentant une AANC. Ce traitement conservateur permet d’éviter la chirurgie et ses complications à plus des deux tiers des patients, tout en sachant que son échec n'augmente pas la morbidité.
